# The analgesic effect of paeoniflorin: A focused review

**DOI:** 10.1515/biol-2022-0905

**Published:** 2024-08-29

**Authors:** Mingzhu Li, Xudong Zhu, Mingxue Zhang, Jun Yu, Shengbo Jin, Xiaoli Hu, Haozhe Piao

**Affiliations:** Department of Integrated Traditional Chinese and Western Medicine Medical Oncology, Cancer Hospital of China Medical University, Liaoning Cancer Hospital & Institute, Shenyang, Liaoning, 110042, P.R. China; Department of General Surgery, Cancer Hospital of China Medical University, Liaoning Cancer Hospital & Institute, Shenyang, Liaoning, 110042, P.R. China; First Clinical College, Liaoning University of Traditional Chinese Medicine, No. 33 Beiling Street, Shenyang, Liaoning, 110032, China; College of Acupuncture and Massage of Liaoning Chinese Traditional Medicine, Shenyang, Liaoning, 110847, P.R. China; Department of Neurosurgery, Cancer Hospital of China Medical University, Liaoning Cancer Hospital & Institute, Shenyang, Liaoning, 110042, P.R. China

**Keywords:** paeoniflorin, analgesic effect, molecular mechanism, monomer of Chinese traditional herbs

## Abstract

Pain has been a prominent medical concern since ancient times. Despite significant advances in the diagnosis and treatment of pain in contemporary medicine, there is no a therapeutic cure for chronic pain. Chinese herbaceous peony, a traditional Chinese analgesic herb has been in clinical use for millennia, with widespread application and substantial efficacy. Paeoniflorin (PF), the main active ingredient of Chinese herbaceous peony, has antioxidant, anti-inflammatory, anticancer, analgesic, and antispasmodic properties, among others. The analgesic effect of PF, involving multiple critical targets and pain regulatory pathways, has been a hot spot for current research. This article reviews the literature related to the analgesic effect of PF in the past decade and discusses the molecular mechanism of the analgesic effect of PF, including the protective effects of nerve cells, inhibition of inflammatory reactions, antioxidant effects, reduction of excitability in nociceptor, inhibition of the nociceptive excitatory neuroreceptor system, activation of the nociceptive inhibitory neuroreceptor system and regulation of other receptors involved in nociceptive sensitization. Thus, providing a theoretical basis for pain prevention and treatment research. Furthermore, the prospect of PF-based drug development is presented to propose new ideas for clinical analgesic therapy.

## 1 Introduction

The most recent International Association for the Study of Pain (IASP) definition of pain is an unpleasant sensory and emotional experience associated with, or resembling that associated with, actual or potential tissue damage [1]. This definition applies to both chronic and acute pain, the distinction between the two relies primarily on the duration of pain, the assessment window, pain frequency, intensity, and classification [2]. Pain has been a considerable challenge for clinicians and patients throughout history [3]. Despite advances in diagnosing and treating pain, chronic pain management remains inadequate. Patients frequently experience extreme discomfort that is difficult to relieve; symptoms such as anxiety, depression, insomnia, and loss of appetite negatively impact their daily lives and overall quality of life [4]. For the current status of pain treatment, common treatments in the World Health Organization’s pain treatment guidelines mainly involve medication in Western medicine, including painkillers and adjuvants, opioids, nonsteroidal anti-inflammatory drugs, central nervous system inhibitors, anticonvulsants, and antidepressants [5]. In addition, psychological therapy is available [6,7]. Pain is often complicated by sleep disturbances, emotional disorders, maladaptive behaviors, and ineffective coping strategies. Therefore, multimodal psychological therapy for chronic pain is a reasonable component of pain treatment [5]. Some treatments, not mentioned in the World Health Organization’s pain treatment guidelines, have gradually been accepted in clinical practice. These include non-invasive surgeries such as nerve block injections, denervation surgery, implantable drug delivery systems, and nerve stimulators. However, this treatment is only used in cases where there is no response to oral and systemic analgesics [8]. Currently, Western medicine treatments have these problems, including central nervous system inhibitors that can damage the patient’s energy, activity, memory, and motor abilities [5]. The common side effects of opioid drugs include sedation, dizziness, nausea, vomiting, constipation, physical dependence, tolerance, and respiratory suppression [9,10]. The uncommon side effects include hyperalgesia, immune and hormonal dysfunction, muscle rigidity, and myoclonus, and opioid drugs can only alleviate pain by an average of 30% [8,11]. Invasive surgical treatment is too expensive; the scope of non-invasive treatment is relatively small [12]. Traditional Chinese medicine believes that there are two causes of pain, one is “pain if not honored,” and the other is “pain if not understood” [13]. So, there are currently traditional Chinese medicine decoctions for the treatment of pain in traditional Chinese medicine. Traditional Chinese medicine doctors can often choose different traditional Chinese medicine formulas corresponding to different syndrome types based on the patient’s pain pathogenesis, including Shaoyao Gancao Tang, which can relieve pain slowly, Fuzi Tang, which can warm meridians and disperse cold, and Huangqi Guizhi Wuwu Tang, which can nourish qi and blood [14]. Acupuncture and moxibustion are also effective for the treatment of pain, especially for local pain such as low back pain or migraine [15]. Traditional Chinese medicine external treatment methods also have good clinical efficacy for pain, including the external application of traditional Chinese medicine, fumigation, and washing of traditional Chinese medicine, and ion introduction of traditional Chinese medicine [16]. All of these traditional Chinese medicines have the characteristics of safety, minimal side effects, and high patient compliance [17]. The Chinese herbaceous peony is a traditional pain-relieving herb whose effectiveness has been recorded as early as the Han dynasty in *Shen Nong Ben Cao Jing*: “It is primarily used for alleviating abdominal pain due to pathogenic factors, removing blood-arthralgia, treating intractable chills and fever, ameliorating hernia, relieving pain, facilitating urination, and benefiting *Qi*” [18]. Its effect of “treating abdominal pain, removing blood arthralgia, tonifying hernia and relieving pain” is the basis of the analgesic effect of paeoniflorin (PF) in this study, and also provides a theoretical basis for the study of the analgesic mechanism of traditional Chinese medicine PF. In the subsequent summary, we mentioned that paeoniflora can relieve spasmodic pain, mainly visceral pain, which also confirmed its effect of “treating abdominal pain, toning hernia and relieving pain”. It was also mentioned that paeoniflora can relieve neuropathic pain (NP), including diabetic peripheral neuralgia and peripheral neuralgia after chemotherapy. In traditional Chinese medicine, Diabetic peripheral neuralgia and peripheral neuralgia after chemotherapy belong to the category of “blood arthralgia,” then it also confirms the role of “removing blood arthralgia” in its efficacy. Therefore, both the efficacy of Chinese medicine and the mechanism of action of modern medicine are interrelated. Chinese herbaceous peonies can be classified as white and red peony roots. According to the 2020 edition of the Chinese Pharmacopoeia, qualitative and quantitative studies revealed the primary chemical component of white and red peony roots to be PF, which can be used as its quality marker [19]. There are some differences between white peony root and red peony root. First, the roots of white peony root and red peony root are different in origin. White peony root mainly comes from cultivated plants in Anhui, Zhejiang, and Sichuan provinces in south China, while red peony root comes from wild plants in Inner Mongolia and northeast China [20]. The difference in origin also leads to the difference in the amount of PF that can be extracted from the two. In Shu Zhu’s study, the quantitative analysis of the components of the two PF samples from northern China found that the PF samples from southern China were derived from the PF samples [21]. Second, the content of PF in stems and leaves at different developmental stages is different. In the study of Tong Ningning et al., it was found that except paeonol and phenolic substances, the content of metabolites of PF and PF in leaves was significantly higher than that in stems at each developmental stage, and the content of PF in leaves was the highest at the first developmental stage (branching stage), higher than the standard stipulated in Chinese Pharmacopoeia. It is the best harvesting stage for medicinal purposes [22]. Finally, the main diseases of the two are different. Paeony has been used to treat dizziness, limb cramps, abdominal pain, diarrhea, blood deficiency, irregular menstruation, and other symptoms. Therefore, PF could be the primary material basis for peonies’ medicinal effects. Further studies revealed that PF has antioxidant, anti-inflammatory, anticancer, and antispasmodic properties, apart from a remarkable analgesic effect [23]. In addition, the synergistic effects of PF make it effective against a wide range of pain. Although this article is mainly written about the analgesic effect of PF, its anticancer, antispasmodic, anti-inflammatory, and antioxidant effects are also of great significance for medical development. PF mainly inhibits the proliferation and migration of cancer cells by inhibiting or activating related pathways [24]. For example, PF can inhibit the proliferation of endometrial cancer cells by activating MAPK and NF-κB signaling pathways [25], and inhibit the proliferation and invasion of breast cancer cells by inhibiting Notch 1 signaling pathway [26]. It also can inhibit Skp2, thereby inhibiting the survival and invasion of liver cancer cells [27]. However, through reviewing the existing literature on the analgesic effect of PF, it can be seen that most of the studies on the analgesic effect of PF are still based on animal experiments, and the research on the molecular mechanism of PF’s analgesic effect has been very mature, but there are very few literatures on the application of PF in human clinical controlled trials. The analgesic effect of PF remains at the mechanism level, and whether it has an exact effect on clinical diseases is still controversial, which is also a challenge and blank in the field of PF research. Based on the incidence of pain in various diseases, the main pain types controlled by PF and the main molecular mechanisms of its analgesic effect were summarized in this paper.

## 2 Extraction of PF

PF is a highly water-soluble monoterpenoid glycoside compound extracted for the first time from *Paeonia lactiflora*. It has also been found in other plants of the Paeoniaceae family, such as Red Peony and Red Peony. As mentioned above, the quality and purity of PF extraction from red peony and white peony are mainly related to the origin, growth stage, and the content of PF in stems and leaves of the two. The most classic method for its extraction is ultrasonic extraction, and the commonly used solvents for this extraction method are methanol or ethanol. For ethanol solution, the concentration of ethanol solution will affect the extraction rate of PF. In the study of Wang Fengqin, it was found that in the process of extracting monoterpene glycosides (mainly PF and its analogs), with the increase of ethanol concentration, the extraction of monoterpene glycosides showed an increasing trend first, and then a decreasing trend, reaching a peak at 30%. This may be due to the fact that the polarity of 30% ethanol is closest to the polarity of monoterpene glycosides, which makes monoterpene glycosides more soluble, while too low or too high polarity may be detrimental to the extraction of monoterpene glycosides [28]. The effect of methanol solvent on the extraction of PF has not been investigated in the literature. In addition to the ultrasonic extraction method, there are also traditional reflux extraction method and solvent-crushing extraction method. However, the reflux extraction method and solvent crushing extraction method have disadvantages such as long analysis time, complicated operation procedure, low selectivity, low extraction efficiency, and need to use a lot of toxic reagents [29]. Ultrasonic extraction of PF has another great advantage. The thermal effect caused by ultrasonic high-frequency vibration may destroy the structure of some monoterpene glycosides and reduce the yield of monoterpene glycosides [30]. When the ultrasonic temperature was lower than 50°C, the extraction rate of monoterpene glycosides increased with an increase in temperature and reached the maximum value when the ultrasonic temperature reached 50°C, indicating that within a certain range, an increase in temperature can accelerate the molecular movement rate, reduce the surface tension of the solvent, increase the solubility of monoterpene glycosides in the solvent, and thus accelerate the dissolution of monoterpene glycosides. When the ultrasonic temperature exceeded 50◦C, the extraction rate of monoterpene glycosides decreased with the increase of temperature, which could be attributed to the destruction of the structure of monoterpene glycosides caused by high temperature. However, if the ultrasonic temperature was controlled within 50°C, the influence of high temperature on the purification of PF would be avoided, which is also the advantage of ultrasonic extraction compared with other high-temperature extraction [28]. Therefore, ultrasonic extraction can be considered a classic extraction method, because it is simple to operate, has high extraction efficiency, and can avoid the instability of monoterpenoid glycosides such as PF caused by high-temperature extraction [31,32]. But at present, in addition to ultrasonic extraction, a promising extraction method has been found in the literature, that is, accelerated solvent extraction (ASE) [33]. ASE is an automatic extraction technology that uses increased temperature and pressure to achieve extraction in a short time, with the advantages of short extraction time, solvent consumption, simultaneous processing of multiple samples, and easy automation. However, ASE has not been found in the extraction of PF, which is the future experimenters can try and expand the field [34]. In summary, to optimize the extraction rate of PF, it is recommended to choose the ultrasonic-assisted extraction method, the concentration of ethanol solvent is controlled at about 30%, and the temperature is controlled at 40–50°C.

## 3 Diverse analgesic effects of PF

Pain can be classified into the following six categories based on its pathophysiological mechanisms: (1) inflammatory; (2) neuropathic; (3) cancer; (4) spastic; (5) psychogenic; and (6) others [36–63]. PF has significant analgesic effects on various pain types [35]. This study summarized the analgesic indications of PF for six categories of pain to understand its rationale for treating them (see Table 1).

**Table 1:** Types of PF analgesia

Types of pain	Related clinical diseases	Possible analgesic mechanisms	References
Inflammatory pain	Rheumatoid arthritis (RA)	Inhibiting fibroblast-like synoviocyte proliferation and cytokine secretion, decreasing IL-1β and TNF-α levels, and regulating mesenteric lymph nodes and Peyer’s patches.	[40,41]
NP	NP caused by nerve damage, diabetes, or chemotherapeutic drugs	NP: inhibiting ASK1 phosphorylation, protecting Schwann cells from demyelination and ER stress, increasing norepinephrine release, activating α2-adrenergic receptors, and regulating spinal cord injury signaling.	[44,45,46,47]
Cancer pain	Tumor-induced pain	Activating p53/14-3-3ζ inhibits tumor cell proliferation, inhibits nuclear factor-κB activation to suppress gastric cancer cell multiplication, and reverses morphine resistance.	[50,51,52,53]
Spasmodic pain	Most visceral pain, Raynaud’s disease, dysmenorrhea, post-surgical smooth muscle spasm	Inhibiting KCl- or ACh-induced smooth muscle contractions.	[56]
Psychogenic pain	Depression accompanied by NP, irritable bowel syndrome, neuroleptic malignant syndrome	Depression accompanied by NP: improving neuroinflammation and providing neuroprotection. Irritable bowel syndrome: inhibiting the leptin/LepRb signaling pathway and reducing PI3K/AKT phosphorylation and BDNF expression.	[59,61]
		NMS: inhibiting nociceptive sensitization.	[63]
Other pain	Muscle soreness and postoperative pain	Postoperative pain: inhibiting the activation of the microglial TLR4/MMP9/2/IL-1b signaling pathway.	

### 3.1 Inflammatory pain

Inflammatory cell infiltration promotes the release of pro-inflammatory cytokines, which induces peripheral and central sensitization of pain [36,37]. PF can exert anti-inflammatory and analgesic effects by inhibiting the release of pro-inflammatory and upregulating the expression of anti-inflammatory factors. Intraperitoneal injection of PF (80 mg/kg) showed good analgesic efficacy in the Feverfew adjuvant-induced inflammatory pain model in mice [38]. Rheumatoid arthritis (RA) is a typical inflammatory disease characterized by joint pain, swelling, and stiffness. PF showed significant anti-inflammatory effects in RA rats and improved their disease resistance [39]. Moreover, inhibition of inflammation and bone erosion in rats with collagen-induced arthritis reduced the proliferation of fibroblast-like synoviocytes and inhibited cytokine secretion, thereby improving RA [40]. Furthermore, PF can accumulate in the intestine and modulate the function of mesenteric lymph nodes and Peyer’s patches, resulting in anti-arthritic effects [41].

### 3.2 NP

Chronic NP, caused by central or peripheral nerve injury, long-term diabetes, or chemotherapeutic drugs, includes trigeminal neuralgia, postherpetic neuralgia, diabetic peripheral neuropathy, post-surgical or traumatic nerve entrapment, and radiotherapy-induced NP [42]. Besides, animal studies showed that PF alleviated NP caused by various etiologies through multiple pathways [43]. By decreasing the expression of phosphorylated p38 (p-p38) and phosphorylated c-jun N-terminal kinase (p-JNK) and inhibiting apoptosis signal-regulating kinase 1 (ASK1) phosphorylation, PF can delay the progression of NP, increase the paw withdrawal threshold and latency, and reduce NP in rats with chronic constrictive injury pain [44]. PF also inhibits paclitaxel-induced mechanical abnormal pain and numbness by increasing circulating peripheral blood flow [45,46]. Furthermore, PF increases norepinephrine release in the spinal cord, activates α2-adrenergic receptors, modulates spinal cord injurious messaging, and alleviates diabetes-induced neuropathy, among others [47].

### 3.3 Cancer pain

PF alleviates cancer pain, primarily caused by local inflammation and nerve damage due to tumor infiltration [48]. Chinese herbaceous peony is the most frequently used herbal medicine for cancer pain [49]; therefore, its analgesic effect is well recognized.

PF simultaneously inhibits the growth and reproduction of cancer cells, thus addressing both the symptoms and root causes. As an effective antitumor agent, PF inhibits tumor proliferation and promotes apoptosis in various tumor cells, such as colorectal cancer, gastric cancer, and glioma [50,51,52]. In addition, PF was known to modulate *N*-methyl-d-aspartate receptor inhibition excitability to reverse morphine resistance in mice [53]. PF can also directly inhibit morphine-induced microglia activation, thereby enhancing morphine acute analgesia and weakening morphine chronic analgesia tolerance [54]. However, there are no clinical studies on PF reversing morphine resistance and reducing morphine chronic analgesia tolerance in human cancer pain. Therefore, this is the limitation of the current research on the analgesic effect of PF on cancer pain. It is hoped that future clinicians can design relevant clinical trials on the basis of this animal experiment, so as to provide clinical data basis for the reversal of morphine resistance and the reduction of morphine chronic analgesic tolerance in the treatment of human cancer pain and provide a new analgesic regimen for the clinical treatment of cancer pain. In addition, PF reverses morphine resistance in patients with cancer pain.

### 3.4 Spasmodic pain

Spastic pain, also known as ischemic pain, is characterized by histopathological changes such as vascular stenosis, tissue ischemia, edema, and dysfunction. Spastic pain includes most visceral pains, Raynaud’s disease, dysmenorrhea, and post-surgical smooth muscle spasms. Spastic abdominal pain-irritable bowel syndrome is one of the patients’ principal challenges and research priorities [55]. PF inhibits KCl or acetyl choline (ACh)-induced contraction of the rectal ring, thus relieving abdominal pain caused by smooth muscle spasms [56].

### 3.5 Psychogenic pain

The human central nervous system can integrate pain sensation with emotional experience, and psychological factors can facilitate pain perception. Therefore, the efficacy of analgesic monotherapy is poor, and the co-morbidity of psychogenic pain and depression is a significant clinical challenge [57]. Bai et al. reported that PF had a satisfactory therapeutic impact on NP-induced depressive behaviors, thus improving the co-morbidity [58]. PF also has preventive and restorative effects on interferon-induced depression and improves interferon-α-induced neuroinflammation [59]. PF has a dual neuroprotective and antidepressant effect in chronic mildly stressed rats by the extracellular signal-regulated kinase (ERK)-cAMP responsive element-binding protein (CREB) pathway [60]. Depression-related mental stress chiefly contributes to visceral pain and nociceptive hyperalgesia in patients with irritable bowel syndrome. PF can alleviate anxiety and irritable bowel symptoms in rats by inhibiting the leptin/leptin receptor (LepRb) signaling pathway and decreasing phosphatidylinositol-3 kinase (PI3K)/Protein kinase B (AKT) phosphorylation and brain-derived neurotrophic factor (BDNF) expression [61].

### 3.6 Other pain

The other pain subgroup includes idiopathic pain, reflex pain (referred pain), and pain caused by non-painful disorders (e.g., hyperhidrosis, sleep disorders) that cannot be classified under the above five categories. PF inhibits c-Fos overexpression in the anterior cingulate cortex and the grey matter surrounding the ventral lateral catheter induced in mice with sciatica pathological pain models, attenuates mechanical, and thermal pain thresholds, alleviates insomnia, and improves somatic discomfort [62]. Furthermore, PF substantially reduces postoperative pain in plantar-incised mice by inhibiting the Toll-like receptor (TLR) 4/matrix metalloproteinase-9/interleukin-1β (TLR4/MMP9/2/IL-1β) signaling in microglia [63].

To sum up, it can be seen that the mechanism of action of PF in the above six kinds of pain has been very clear, but whether pf can relieve pain or treat pain is based on animal experiments, which cannot support the efficacy of PF in the treatment of human pain. Therefore, it is suggested that clinical controlled trials should be designed between PF and standard analgesic drugs in the treatment of these six kinds of pain in the future, to explore the efficacy of PF’s analgesic effect, and to study the differences between PF and known pain treatment methods, so as to learn from each other and propose new treatment strategies for pain treatment. The discussion of clinical efficacy can also verify the correct mechanism of action of animal experiments and combine animal experimental studies with clinical trials to study more possible treatment methods for pain.

## 4 Primary analgesic mechanisms of PF

The main analgesic mechanisms of PF include nerve cell protection, inhibition of the inflammatory response, and modulation of the nociceptive-associated neurotransmitter-receptor system [19,38,40,44,58,62,64–102] (see Table 2).

**Table 2:** PF-related analgesic mechanisms

Analgesic mechanisms	The dose of PF	The object of action	Specific effects	References
Nerve-cell protective effect	5 μL of 0.5, 1, 2 μg	Male C57BL/6J mice	a. Inhibiting the activation of microglia and astrocytes	[38,66]
	10 mg/kg; 50, 200 μmol/L	Male SD rats, SC	b. Activating SCs to repair damaged nerves	[69,70,71]
Inhibition of inflammatory response	25, 50, 100 mg/kg	Male SD rats	a. Inhibiting NLRP3 inflammasome activation	[73]
	5, 10, 20 mg/kg; 2.5, 12.5, 62.5 g/mL	Male SD rats	b. Reducing PG release rand COX-2 expression	[40,74]
Antioxidant	Not mentioned	Not mentioned	a. Upregulating Trx2 to enhance mitochondrial function and attenuating mitochondrial damage-induced neural demyelination in SCs	[75]
	50 mg/kg	Female C57BL/6 mice	b. Activating the Akt/Nrf2/Gpx4 pathway to prevent iron death	[76]
Reduction in nociceptor Excitability	Not mentioned	Not mentioned	a. Inhibiting the activation of nociceptor TRPV1	[78,79]
	1,5,25 mg/kg	Male C57BL/6mice	Reducing IL-1β, IL-6, and TNF-α levels, intracellular Ca levels, and PKC activity, and inhibiting TRPV-1 expression, NF-κB transcription, and NLPR3	[80]
Inhibition of the nociceptive excitatory neuroreceptor system	180 mg/kg	Female SD rats	a. Inhibiting the glutamate-receptor system	[82,83,84]
	100 μM	Male 6e8w Balb/c mice	b. Inhibiting the bridging of pain transduction pathways by nuclear factor and nociceptive excitatory neuroreceptor system	[85,86,87,88,89,90]
Activation of the nociceptive inhibitory neuroreceptor system	50, 100 mg/kg	Male SPF C57BL/6j mice	Activating A1Rs	[62,83]
Regulation of other receptors involved in nociceptive sensitization	Not mentioned	Not mentioned	a. Inhibiting cannabinoid CB2 receptor expression	[91,92]
	25, 50, 100 mg/kg	Male Balb/c mice, male SD rats	b. Inhibiting TLR4	[19,58,73,93]
	20, 30, 40, 60 mg/kg	Male SD rats	c. Regulating the MAPK signaling pathway	[44,60,83,94,95,96,97,98,99,100,101,102]

### 4.1 Nerve cell protective effects of PF

#### 4.1.1 PF inhibits the activation of astrocytes and microglia

Astrocytes, the most numerous glial cells in the central nervous system, stabilize the internal environment in the central nervous system [64]. In pathological pain, activated astrocytes can release numerous substances, such as cytokines, prostaglandins (PGs), inflammatory mediators, chemokines, adenosine, and excitatory amino acids, leading to persistent nociceptive thermal and mechanical sensitization [65]. When spinal cord nerve injury occurs, after the behavioral hyperalgesia of spinal cord microglia, the depletion of microglia leads to the disappearance of pain hypersensitivity [66]. PF additionally alleviates inflammatory pain by inhibiting microglial activation and Akt-NF-κB signaling in the central nervous system [38].

#### 4.1.2 PF repairs damaged nerves by activating Schwann cells (SCs)

SCs are the primary glial cells of the nervous system, which can promote axon regeneration [67]. PF activates SCs through multiple pathways to exert neuroprotective effects. The ER is an important cellular organelle that plays a key role in protein folding and calcium dynamics. Increased Ca^2+^ production may interfere with protein folding, leading to an increase in misfolded proteins in the ER. Overload of this folding process can lead to ER stress, which is enhanced in the nervous system under various pain states [68]. Especially in NP conditions. In related studies, it was found that PF reduced cytoplasmic Ca^2+^ in SCs, which would alleviate endoplasmic reticulum stress and NP, thereby reducing paclitaxel-induced mechanical pain in C57BL/6NCr mice [69]. In this study, it was found that PF increased the expression of sciatic nerve thioredoxin 2 (Trx2) and thioredoxin reductase 2 (TrxR2) in DPN rats, thereby improving SC mitochondrial function, inhibiting axon regeneration, reducing sciatic nerve demyelination, increasing mechanical and thermal pain threshold, movement nerve conduction velocity, and sensory nerve conduction velocity, thus providing neuroprotection to relieve diabetic peripheral neuropathy pain. PF also alleviates chronic sciatica by reducing the levels of inflammatory factors such as IL-1, IL-6, and tumor necrosis factor-α (TNF-α) and inhibits axon regeneration by inhibiting the apoptosis of SCs, thus promoting the repair of damaged nerves [70]. Similarly, PF inhibits hydrogen peroxy-induced SC injury and apoptosis by inhibiting the phosphorylation of p38 mitogen-activated protein kinase (MAPK) and reducing the levels of caspase3, cleaved caspase3, and cleaved caspase7, thereby relieving pain. In summary, it can be seen that PF is of great significance for the relief of NP [71].

### 4.2 Anti-inflammatory effect

PF exerts an anti-inflammatory effect by inhibiting the activation of inflammatories and the production of inflammatory mediators in a variety of ways besides the above pathways, so as to achieve an analgesic effect [72]. However, its role in chronic and acute pain is different, such as inhibiting NLRP3 inflammasome activation to relieve chronic sciatica and decreasing COX-2 expression to relieve pain in the acute phase of adjuvant arthritis. The specific functions are as follows.

#### 4.2.1 PF inhibits NOD-like receptor protein 3 inflammasome activation

Activation of the NLRP3 inflammasome mediates the development of NP after chronic systolic injury of the sciatic nerve. PF can reduce sciatic nerve contraction by inhibiting the activation of the NLRP3 inflammasome, thereby reducing NP [73].

#### 4.2.2 PF reduces PG release and COX-2 expression

PGs are important mediators of inflammation and pain relief. PF inhibitors the production of TNF-α inflammatory factors in serum macrophages Q9, IL-1, and advanced arthritis (AA) rates, while PGE2 can also reduce the production of inflammatory mediators and executive administrative effects [74]. Cyclooxygenase (COX), also known as PG endooxygenase reductase, is an essential enzyme for the synthesis of various PGs. Excessive secretion of PGs can stimulate frequent muscle contractions and cause pain (literature). Inhibiting COX-2 is an important mechanism for PF analgesia. PF inhibits the abnormal proliferation of synovial cells, inhibits the production of IL-1, IL-6, vascular endothelial growth factor (VEGF), PGE2, and granulocyte macrophage colony-stimulating factor (GM-CSF) by synovial cells, and reduces the expression of guanine nucleotide-binding protein (G protein) and COX-2 in synovium. Lowering the expression of COX-2 will prevent it from synthesizing various PGs, resulting in analgesic effects [40].

### 4.3 Antioxidant effect

The neuroprotective effect of PF on activating SCs is related to its antioxidant activity, and Trx2 exerts antioxidant stress while maintaining mitochondrial function. In SCs, PF upregulates Trx2 to enhance mitochondrial activity and attenuates neural demyelination caused by mitochondrial damage, thus alleviating diabetic peripheral NP [75]. Furthermore, the antioxidant activity of PF offers in vitro neuro-cytoprotection by enhancing the anti-lipid peroxidation activity of glutathione peroxidase 4 (Gpx4) and activating the Akt/nuclear factor erythroid 2-related factor 2 (Nrf2)/Gpx4 pathway to prevent FN (fibronectin) [76].

### 4.4 Reduction in nociceptor excitability

Transient receptor potential cationic channel subfamily V Member 1 (TRPV1) is strongly expressed in the mammalian peripheral nervous system and is involved in a variety of physiological and pathophysiological processes. Its activity is important in transmitting pain signals from peripheral sensory neurons to the central nervous system [77]. Specifically, TRPV1 opens a heat-sensitive ion channel upon activation, and subsequently, nerve endings release neuropeptides and excitatory amino acids, inducing pain formation in the cerebral cortex [78,79]. In addition, TRPV1 is closely associated with immune processes and inflammatory signaling pathways in several organs or systems. In related experiments, it was found that PF reduced the transmission of pain signals from peripheral sensory neurons to the central nervous system by inhibiting the expression of TRPV1 and the production of inflammatory factors (IL-1β, IL-6, and TNF-α), resulting in the failure of the cerebral cortex to form pain, thus improving inflammation and pain symptoms in mice [80]. Antidepressants such as duloxetine and amitriptyline, on the other hand, reduce NP by activating α1 and α2 adrenergic receptors that inhibit primary afferent input to the spinal cord [81].

### 4.5 Inhibition of the nociceptive excitatory neuroreceptor system

#### 4.5.1 Inhibition of the glutamate-receptor system

**Figure 1:**
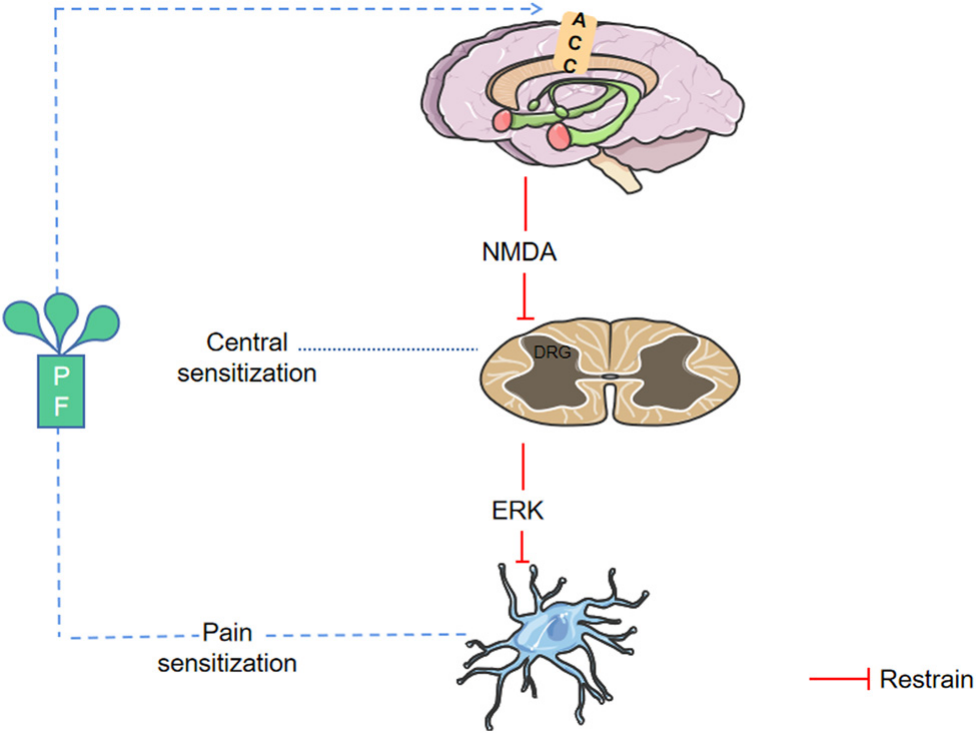
PF induces analgesia by downregulating glutamate levels in ACC and decreasing ERK expression in DRG. Note: PF: paeoniflorin; ACC: anterior cingulate cortex; DRG: dorsal root ganglion; ERK: extracellular signal-regulated kinase. PGE: prostaglandin E; NMDA: *N*-methyl-d-aspartic; β-EP: β-Endorphin; CCL2: chemokine ligand 2.

Glutamate, the most abundant, extensively distributed, and potent excitatory neurotransmitter in the central nervous system, is a crucial factor that induces the persistent development of chronic pain. Pain stimulation induces a dynamic rise in glutamate levels in the brain’s anterior cingulate cortex (ACC) that correlates strongly with the intensity of subjective pain experienced by the participants [82]. *N*-Methyl-d-aspartic acid (NMDA) receptors, an ionotropic glutamate receptor subtype, are implicated in processing acutely injurious visceral input signals. Increased NMDA receptor-mediated excitatory synaptic transmission in dorsal horn neurons can result in nerve injury-induced central sensitization. When measuring glutamate concentrations in microdialysis solutions from awake rats using capillary electrophoresis combined with laser-induced fluorescence detection, researchers found that PF reversed the CRD-induced increase in ACC glutamate concentrations, resulting in an analgesic effect [83]. NMDA can activate the ERK/CREB signaling pathway, triggering intracellular changes. PF can produce analgesia by activating adenosine A1 receptors (A1Rs), inhibiting glutamate release and suppressing CRD-induced NMDA receptor-dependent ERK signaling [84] (see Figure 1).

#### 4.5.2 Inhibition of the bridging of pain signal transduction pathways by nuclear factor NF-κB

NF-κB, a transcriptional regulator with pleiotropic regulatory functions, governs the biological activities of multiple upstream and downstream pain-related factors, thus bridging both pain transduction pathways. NF-κB and cytokines have a positive feedback relationship. NF-κB activation upregulates gene expression of TNF-α and IL-1β; this is mediated by the release of inflammatory factors that reactivate NF-κB [85].

PF alleviates neuropathic and inflammatory pain by inhibiting NF-κB expression, Besides, PF relieves NP due to chronic constriction injury of the sciatic nerve (CCI) by inhibiting microglia overactivation and decreasing the expression levels of p-p38 MAPK and NF-κB in the spinal cord [86]. PF acts on the AKT/NF-κB pathway and inhibits the synthesis of inflammatory factors by microglia in the spinal cord, thereby alleviating inflammatory pain [38]. Furthermore, PF can improve RA by inhibiting the NF-κB signaling pathway [87,88].

Intercellular adhesion molecule-1 (ICAM-1), primarily expressed in endothelial and immune cells, is instrumental in leukocyte migration and activation. PF inhibits monocytic lipopolysaccharide-induced ICAM-1 expression by suppressing the NF-κB signaling pathway [89]. PF suppresses the inflammatory activity of B cells by downregulating the NF-κB/ERK signaling pathway. Inducible nitric oxide synthase (iNOS) has neurotoxic effects. By inhibiting the NF-κB signaling pathway in activated macrophages, PF inhibits the expression of nitric oxide and iNOS, thus offering neuroprotection [90].

### 4.6 Activation of the nociceptive inhibitory neuroreceptor system

#### 4.6.1 Activation of A1Rs

A1Rs are widely expressed in the brain. Their activation inhibits the appetitive, histaminergic, and cholinergic awakening neurons, improving non-rapid eye-movement sleep (NREM) [62]. PF induces analgesia and sleep improvement by acting on A1Rs and inhibiting p-ERK and c-Fos expression in the lumbosacral dorsal horn; thus, A1R antagonists can block the analgesic effect of PF, suggesting that PF can relieve pain by activating adenosine A1 to inhibit the extracellular signaling ERK pathway [83]. Mechanical threshold, thermal latency, and NREM were not altered by PF in A1 knockout mice, further substantiating the analgesic and hypnotic action of PF mediated via adenosine A1 [62].

### 4.7 Regulation of other receptors involved in nociceptive sensitization

#### 4.7.1 Inhibition of cannabinoid receptor 2 (CB2) expression

CB2, a G protein-coupled receptor, is expressed primarily on immune cells in the blood and peripheral tissues and minimally on neurons and glial cells [91]. Long-term morphine administration induces a time-dependent upregulation of CB2 receptor expression in the spinal cord of animals, indicative of CB2 possibly mediating morphine analgesic tolerance [92].

#### 4.7.2 Inhibition of TLR4

TLRs are an important class of protein molecules involved in nonspecific immunity that recognize microbial-conserved structures. TLR4 could modulate pain by inducing downstream inflammatory factors, while elevated TLR4 expression in neuropathic mouse microglia could induce nociceptive hyperalgesia [93]. PF ameliorates NP-induced depression-like behavior in mice by inhibiting TLR4/NF-κB pathway-activated hippocampal neuroinflammation [58]. Furthermore, PF suppresses postoperative pain by modulating the microglia’s TLR4/MMP9/2/IL-1β signaling pathway [19]. PF inhibits apoptosis and inflammatory infiltration of SCs by decreasing caspase3 and TLR4 levels in sciatic nerve tissue, thereby improving NP in rats with CCI [73].

#### 4.7.3 Regulation of the MAPK signaling pathway

MAPK protein activation is necessary for inflammation, neural injury-induced NP development, and peripheral and central sensitization [94]. The three primary members of the MAPK family, ERK, p38 MAPK, and JNK, are activated primarily in neurons, microglia, and astrocytes, respectively [95,96].

The p38 MAPK pathway can be activated by multiple inflammatory factors. It is activated as p-p38 MAPK, contributing to the development and maintenance of pain [97,98]. p-p38 MAPK aids in the translational synthesis of several inflammatory factors, lowering the nociceptive threshold of neurons and inducing NP [99,100]. Moreover, JNK is a vital kinase mediating astrocyte activation and chronic pain. ASK1, an upstream protein of p38 and JNK, is involved in the induction and maintenance of chronic pain [101]. PF improves NP by inhibiting ASK1 phosphorylation and decreasing p-p38 and p-JNK expression [44]. The ERK-CREB signaling pathway is critical in developing and maintaining NP. NMDA receptor-dependent ERK activation in the spinal cord contributes to the establishment and prolonged maintenance of nociceptive hyperalgesia [102]. PF-activated A1Rs inhibit NMDA receptor-dependent ERK signaling induced by colorectal distension (CRD) in rats and alleviate CRD-induced visceral hyperpathia [83].

**Figure 2:**
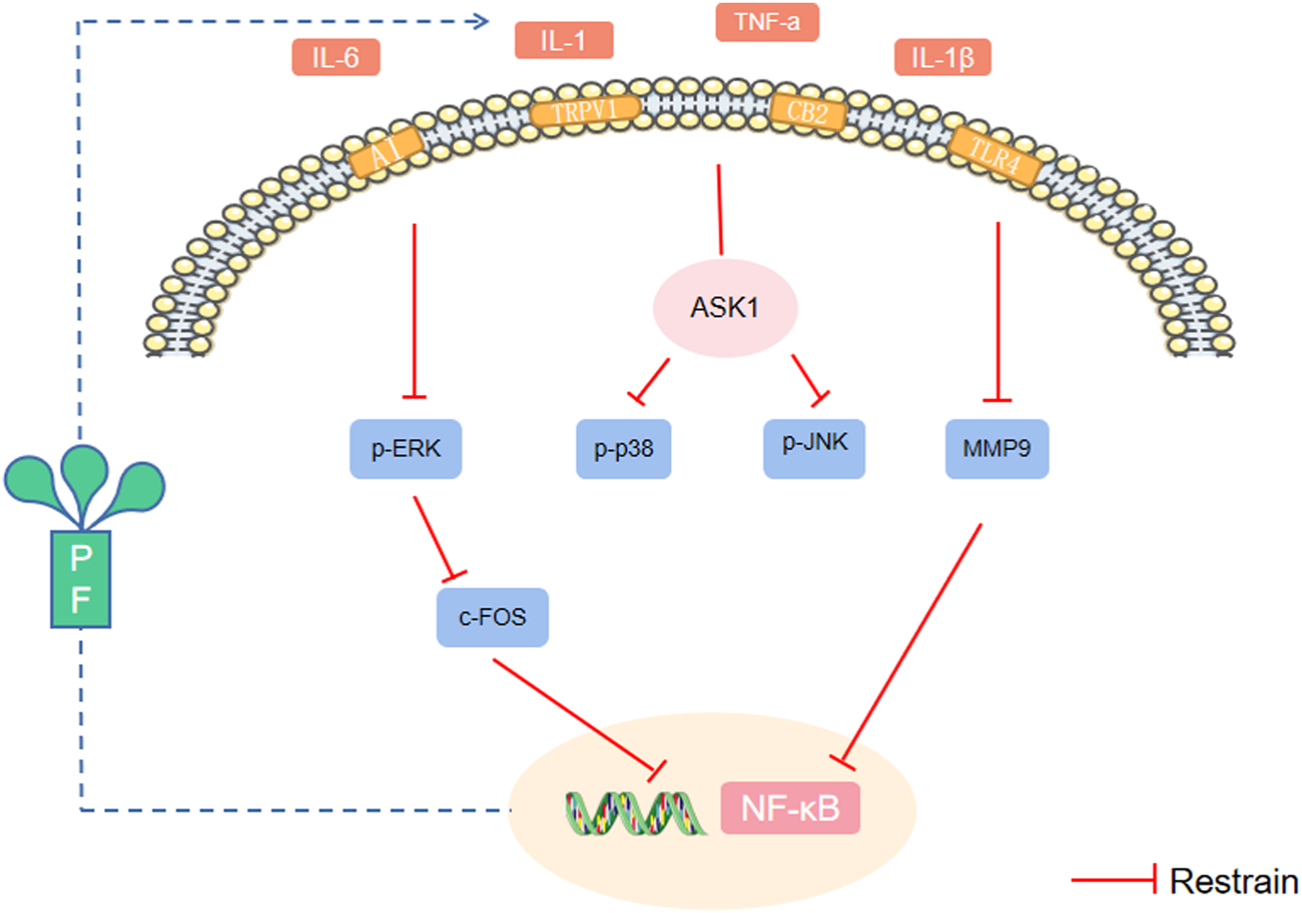
The analgesic effect of PF is mediated through the regulation of MAPK and upstream inflammatory factors. Note: MAPK: mitogen-activated protein kinases; NF-κB: nuclear transcription factor-κB; ASK1: apoptosis signal-regulated kinase 1; p-ERK: p-extracellular signal-regulated kinase; p-p38: phosphorylated-p38; p-JNK: phosphorylated c-jun N-terminal kinase; MMP9: metalloproteinase-9; c-FOS: protooncogene; TNF-α: tumor necrotic factor-α; IL-1β/IL-1: interleukin-1; IL-6: interleukin-6.

Numerous reports have indicated that PF attenuates NP in rats with chronic constriction injury by inhibiting the MAPK signaling pathway. New rat studies suggest that PF protects the hippocampal neurons from chronic unpredictable mild stress (CUMS)-induced damage by activating the ERK-CREB signaling pathway. The neuroprotective and antidepressant effects of PF in CUMS rats were inhibited by blocking the ERK-specific blocker U0126, possibly owing to the distinct effects of PF on the ERK-CREB pathway in the hippocampus and spinal cord. Therefore, PF may exert complex neuroprotective and analgesic effects at different sites (see Figure 2).

## 5 Conclusion

Poor outcomes in chronic pain management are a growing challenge for clinicians. The risk of abuse, abuse, and addiction to the most commonly used opioid painkillers, even at prescribed doses, can be fatal and is a serious public health concern. In addition to addiction, traditional opioid analgesics can also cause side effects such as dizziness, nausea, vomiting, constipation, and respiratory depression, which affect the quality of life of patients. Now, the top priority for pain management is to find drugs that have fewer side effects than traditional opioids and can effectively relieve patients’ pain. The Chinese herbal medicine Peony is a traditional painkiller, and its active ingredient PF can effectively relieve inflammation, neuropathic, cancer, and psychogenic pain, and the mechanism of its analgesic effect has been confirmed in animal experiments. PF can therefore be used to develop potential non-opioid painkillers for the treatment of chronic pain. The main mechanisms of PF analgesia are as follows: (1) inhibit the release of inflammatory and immune-related factors such as IL-1, IL-1β, IL-6, TNF-α, NLRP and PGs; (2) limit ROS levels to exert antioxidant effects; (3) PF achieves immune-related effects by inhibiting the activated NF-κB signaling pathway in macrophages; (4) regulation of pain related signaling pathways such as MAPK, TRPV1, TLR, and TOLL. Thus, by inhibiting nociceptive sensitization, PF can modulate the neuroprotective role of SCs, astrocytes, and microglia and subsequently relieve pain symptoms. Based on the above literature review, we found that the analgesic mechanism of PF has been very mature at present, but there are still three problems that need our attention. First, in animal studies of PF’s analgesic effects, there are no criteria for detecting pain behavior. For the detection of pain behavior in rats or mice, some researchers use mechanical pain hypersensitivity testing, while others use cold and hot pain hypersensitivity testing. There are many methods for detecting mechanical pain hypersensitivity, including Von Frey fiber method, Randall foot pressure test, etc. There are also many methods for detecting cold and hot hyperalgesia, including acetone test, cold water immersion test, cold foot test, hot radiation mouse tail test, hot plate test, etc. If there is no unified method for detecting pain behavior, it will greatly affect the results of mechanism research. Therefore, it is suggested that future experimenters can establish a unified and standardized method for pain behavior detection, so as to provide more accurate and rigorous experimental data for basic research on pain, and also provide a solid foundation for clinical research. Secondly, the current research on the extraction and purification of peony is limited, and some researchers did not mention the concentration of peony in the literature. There is also the current extraction method of PF is too simple, most of them are ultrasonic extraction, which leads to the extraction rate of PF in the experiment being very different, and there is indeed a more promising ASE method than ultrasonic extraction, but it has not been applied in the extraction of PF. Therefore, it is suggested that future experimenters should try more new extraction methods, perhaps there will be a higher purity of PF used in experimental studies, then there may be more pathways about PF analgesic mechanism to be explored. As a clinical hospital, our goal is to use PF to address the symptoms of pain patients and improve their quality of life. Moreover, the harm of pain to patients has been mentioned in the introduction, as well as the limitations of current opioid analgesics in the treatment of pain. Therefore, to prove the potential value of PF as a non-opioid analgesic in pain management, the results of clinical trials are required. However, all current studies are limited to animal experiments. Therefore, it is very necessary and urgent to conduct PF clinical trials in time. Therefore, it is recommended that future clinicians design clinical controlled trials of PF in different types of pain (such as inflammatory pain, NP, and cancer pain) to determine the efficacy of paeoniflora in different types of pain, and clinical studies can also determine and continuously optimize the administration method and dose of PF analgesia according to different pain types. The efficacy of PF alone and PF combined with other drugs were compared through controlled trials, so as to further provide new diagnosis and treatment ideas for pain patients.
